# Brain connectomic associations with traditional Chinese medicine diagnostic classification of major depressive disorder: a diffusion tensor imaging study

**DOI:** 10.1186/s13020-019-0239-8

**Published:** 2019-04-11

**Authors:** Lan-Ying Liu, Xiao-Pei Xu, Li-Yuan Luo, Chun-Qing Zhu, Ya-Ping Li, Pei-Rong Wang, Yuan-Yuan Zhang, Chun-Yu Yang, Hong-Tao Hou, Yu-Lin Cao, Gang Wang, Edward S. Hui, Zhang-Jin Zhang

**Affiliations:** 1 Department of Psychiatry, Tongde Hospital of Zhejiang Province, Hangzhou, 310012 Zhejiang China; 2 Department of Diagnostic Radiology, LKS Faculty of Medicine, The University of Hong Kong, Hong Kong, China; 3 Department of Internal Chinese Medicine, Tongde Hospital of Zhejiang Province, Hangzhou, 310012 Zhejiang China; 4 Department of Radiology, Tongde Hospital of Zhejiang Province, Hangzhou, 310012 Zhejiang China; 5 School of Chinese Medicine, LKS Faculty of Medicine, The University of Hong Kong, 10 Sassoon Road, Pokfulam, Hong Kong, China

**Keywords:** Major depressive disorder, Traditional Chinese medicine, Classification, Diffusion tensor imaging, Connectome

## Abstract

**Background:**

Major depressive disorder (MDD) is highly heterogeneous in pathogenesis and manifestations. Further classification may help characterize its heterogeneity. We previously have shown differential metabolomic profiles of traditional Chinese medicine (TCM) diagnostic subtypes of MDD. We further determined brain connectomic associations with TCM subtypes of MDD.

**Methods:**

In this naturalistic study, 44 medication-free patients with a recurrent depressive episode were classified into liver qi stagnation (LQS, n = 26) and Heart and Spleen Deficiency (HSD, n = 18) subtypes according to TCM diagnosis. Healthy subjects (n = 28) were included as controls. Whole-brain white matter connectivity was analyzed on diffusion tensor imaging.

**Results:**

The LQS subtype showed significant differences in multiple network metrics of the angular gyrus, middle occipital gyrus, calcarine sulcus, and Heschl’s gyrus compared to the other two groups. The HSD subtype had markedly greater regional connectivity of the insula, parahippocampal gyrus, and posterior cingulate gyrus than the other two groups, and microstructural abnormalities of the frontal medial orbital gyrus and middle temporal pole. The insular betweenness centrality was strongly inversely correlated with the severity of depression and dichotomized the two subtypes at the optimal cutoff value with acceptable sensitivity and specificity.

**Conclusions:**

The LQS subtype is mainly characterized by aberrant connectivity of the audiovisual perception-related temporal-occipital network, whereas the HSD subtype is more closely associated with hyperconnectivity and microstructural abnormalities of the limbic-paralimbic network. Insular connectivity may serve a biomarker for TCM-based classification of depression.

*Trial registration* Registered at http://www.clinicaltrials.gov (NCT02346682) on January 27, 2015

**Electronic supplementary material:**

The online version of this article (10.1186/s13020-019-0239-8) contains supplementary material, which is available to authorized users.

## Background

Major depressive disorder (MDD) is a serious mental illness that affects 8–20% of the worldwide population [1]. Although various pharmacological and non-pharmacological therapies have been developed, the treatment outcomes are unsatisfactory [2]. This is largely because MDD is a complicated condition with multi-system etiopathogenesis and diverse clinical manifestations. In addition to the core symptoms of low mood, loss of interest, and pleasure, MDD is often comorbid with somatic and other psychiatric symptoms, such as pain, sleep disturbance, anxiety, and neurocognitive dysfunction [3]. It is thought that further classification could help characterize its symptomatic and pathological heterogeneity. Although various classification approaches of MDD have been explored, there is still a dearth of compelling evidence for the existence of depressive symptom dimensions and symptomatic subtypes [4]. Therefore, the development of a novel classification system that could improve clinical applicability and more precisely differentiate MDD subtypes is highly desired.

Traditional Chinese medicine (TCM) is an ancient medical practice which has established a distinct diagnostic system. One key diagnostic methodology is to differentiate etiopathological patterns on the basis of clinical symptoms and signs collected through inspection, auscultation, olfaction, interrogation, and palpation of the pulses [5]. According to TCM theory, depressive disorders can be classified into at least six different patterns [6–8]. Among them, liver qi stagnation (LQS) and heart and spleen deficiency (HSD) subtypes are the two most commonly occurring and opposing patterns, accounting for approximately 2/3 of depressed patients [6–8]. The diagnostic criteria for the two patterns have been well validated by several large-scale studies as shown in Table 1 [6–8]. The LQS subtype is often comorbid with nervousness and irritability; whereas the HSD subtype is characterized by excessive pensiveness, suspicion, and timorousness. One study has revealed considerable differences in the functional connectivity of the posterior cingulate cortex between “deficiency” and “excessive” patterns of MDD [9]. Most recently, we have revealed differential metabolomic profiles of the LQS and HSD subtypes of MDD [10]. These findings have encouraged us to further explore brain connectomic correlates of TCM-based subtypes of MDD.

**Table 1:** Clinical manifestations and diagnostic criteria of TCM-based subtypes of MDD

	Liver qi stagnation (LQS)	Heart and spleen deficiency (HSD)
Mood symptom characters	Depressed mood with frustration, nervousness, and/or irritability	Depressed mood with excessive pensiveness, suspicion, and/or timorousness
Somatic symptoms	A. Frequently sighing (0 = absent, 1 = slight, 2 = mild, 3 = moderate, 4 = severe) B. Chest distension and/or hypochondriac pain (0 = absent, 1 = slight, 2 = mild, 3 = moderate, 4 = severe) C. Abdominal bloating (0 = absent, 1 = slight, 2 = mild, 3 = moderate, 4 = severe) D. Decreased appetite (0 = absent, 1 = mild, 2 = moderate, 3 = severe) E. Loose stool (0 = absent, 1 = mild, 2 = moderate, 3 = severe) F. Breast tenderness (0 = absent, 1 = slight, 2 = mild, 3 = moderate, 4 = severe) G. Irregular menstruation (0 = normal, 1 = seldom, 2 = sometimes, 3 = frequent)^a^ H. Menstrual pain (0 = absent, 1 = mild, 2 = moderate, 3 = severe)^a^	A. Palpitation (0 = normal, 1 = seldom, 2 = sometimes, 3 = most times, 4 = all times) B. Forgetfulness (0 = absent, 1 = slight, 2 = mild, 3 = moderate, 4 = severe) C. Insomnia or dream-disturbed sleep (0 = absent, 1 = sometimes, 3 = most times) D. Decreased appetite (0 = absent, 1 = slight, 2 = mild, 3 = moderate, 4 = severe) E. Abdominal fullness (0 = absent, 1 = sometimes, 3 = most times) F. Loose stool or dysfunctional diarrhea (0 = absent, 1 = slight, 2 = mild, 3 = moderate, 4 = severe) G. Pale and sallow complexion (0 = normal, 1 = slightly apparent, 2 = mildly apparent; 3 = moderately apparent, 4 = very apparent) H. Tiredness (0 = absent, 1 = mild, 2 = moderate, 3 = severe)
Tongue and pulse	I. Red tongue body with thin and white coating (0 = normal, 1 = mildly apparent, 2 = moderately apparent, 3 = very apparent) J. Wiry pulse (0 = normal, 1 = slightly apparent, 2 = mildly apparent; 3 = moderately apparent, 4 = very apparent)	I. Pale and tender or watery tongue body with white coating (0 = normal, 1 = slightly apparent, 2 = mildly apparent; 3 = moderately apparent, 4 = very apparent) J. Weak and thin pulse (0 = normal, 1 = slightly apparent, 2 = mildly apparent; 3 = moderately apparent, 4 = very apparent)
Diagnostic criteria^b^	[1] Must have A, B, I, and J; at least one of D and E; and at least one of F, G, and H for women [2] Total score of physical symptoms and tongue and pulse signs is not less than 10	[1] Must have at least two of A, B and C; at least two of D, E and F; at least one of G and H; I and J [2] Total score of physical symptoms and tongue and pulse signs is not less than 10

The diagnostic criteria are modified based on Refs. [6–8]

^a^F, G, and H items are only applied for women

^b^Those who fail to meet either LQS or HSD subtype are classified as other subtypes

Structural magnetic resonance imaging (MRI) studies have well demonstrated microstructural white matter abnormalities in distributed brain regions of depressed patients [11–13]. Significant differences in loss of white matter integrity also have been observed between atypical and melancholic subtypes of MDD [14] and between bipolar I and bipolar II disorder [15]. These studies suggest that white matter network connectivity may be potential biomarkers for differentiating subtypes of MDD.

On the basis of our metabolomic findings [10], we hypothesized that there also exist different connectomic profiles of the LQS and HSD subtypes of MDD. To test this hypothesis, diffusion tensor imaging (DTI)-based whole-brain network analysis, also known as connectomics, was conducted to compare structural white matter interregional connectivity and microstructural integrity in the LQS and HSD subtypes of depressed patients and healthy subjects, most of whom had participated the previous metabolomic study [10].

## Materials and methods

### Setting and participants

This naturalistic study was a parallel investigation of our previous metabolomic study [10], which was carried out at Tongde Hospital of Zhejiang Province in Hangzhou, China between April 2015 and March 2017. The study protocol was approved by the Medical Ethical Committee of Tongde Hospital and registered at www.clinicaltrials.gov (NCT02346682). All participants gave voluntary, written, informed consent before entering the study. We reported this study according to the Minimum Standards of Reporting Checklist (see Additional file 1).

Subjects were eligible for this study if they: (a) were aged 18–65 years; (b) were currently experiencing a recurrent, moderate or severe depressive episode according to the Diagnostic and Statistical Manual of Mental Disorders, Fifth Edition (DSM-5), as evidenced by a score of at least 21 on the 24-item Hamilton Rating Scale for Depression (HAMD-24) [16]; (c) met the diagnostic criteria of the LQS or HSD subtype as defined in Table 1; (d) had received no treatment with antidepressants or other psychotropic drugs in the previous 3 months; and (e) were right-handed.

Patients were excluded from this study if they: (a) were experiencing their first episode of depression; (b) had serious comorbid cardiac, hepatic, or renal conditions; (c) had a history of brain injury or surgery; (d) had a history of manic, hypomanic, or mixed episodes; (e) had received investigational drug treatment within the previous 6 months; (f) had experienced alcohol or drug abuse within the previous 12 months; or (g) were pregnant or breastfeeding.

A group of healthy volunteers who had no personal or family history of significant mental and physical illness was recruited to serve as controls.

### Screening, assessment, and TCM diagnosis

Screening was done by a psychiatrist and a TCM practitioner. The severity of the depression symptoms of the patients and healthy controls was assessed using HAMD-24 [16]. The assessment was conducted by a trained rater (L.Y.L., C.Q.Z.). TCM subtypes diagnoses were made by at least two senior TCM practitioners (Y.P.L., C.Q.Z.) and a third TCM practitioner (L.Y.L.) was involved in the diagnosis process if the first two practitioners could not reach an agreement.

To ensure consistency of assessment and diagnosis, a manual was provided and a training workshop was carried out on videotaped patients with different TCM subtypes before the study was initiated. An inter-rater reliability coefficient of > 0.85 was achieved after the completion of the training workshop.

### Imaging acquisition and preprocessing

To avoid the influence of different coils on the image acquisition, all subjects were scanned in the same scanner (3.0-Tesla, Magnetom Trio Tim, Siemens) with the same 16-channel head coil in the Department of Radiology at Tongde Hospital. The subjects were instructed to keep their eyes closed and their mind relaxed during the scan. A homogeneous birdcage head coil fitted with foam pads was applied to each subject’s head to limit head movement. High-resolution three-dimensional structural images were acquired by using a T1-weighted gradient echo sequence at a voxel size of 1 mm^3^, parallel to the anterior commissure–posterior commissure (AC–PC) line and covering the whole brain. Diffusion-weighted images along 30 gradient directions with a b-value of 1000 were acquired using a single-shot, echo-planar sequence with the following parameters: repetition time = 8600 ms, echo time = 92 ms; flip angle = 90°; number of transversal slices = 55; gradient directions = 30; slice thickness = 2.0 mm without gap; field of view = 256 × 248 mm^2^.

The imaging data obtained were preprocessed and analyzed using statistical parametric mapping (SMP8) package (Wellcome Department of Cognitive Neurology, London), running under Matlab 7.6 (Mathworks, Natick, MA). According to the standard preprocessing procedure, the individual MRI data was first realigned and resliced to 2 × 2 × 2 mm^3^ isotopic voxels. Images of the subject’s head movement ≥ 5 mm in any direction within one session were excluded. Its realigned mat file was applied to spatially normalize all the individual images into a standard Montreal Neurological Institute (MNI) template. The normalized images were then spatially smoothed using a full width half maximum (FWHM) three dimensional Gaussian kernel of 8 mm.

### Brain network construction

Network construction and connectivity analysis were conducted as we have done previously [17]. To determine the brain regions in the network, an automated anatomical labeling (AAL) template was used to obtain 90 cortical and subcortical regions (cerebellum excluded). Masks for each of the regions of interest (ROIs) were transformed into individual subjects’ native spaces in following steps. The subject’s structural image was first registered to his/her DTI image using the affine transform. The native space structural image was then registered to the ICBM-152 brain template in the Montreal Neurological Institute (MNI) space by using the non-linear transformation. The inverse transformation matrix was applied to the atlas and brought ROIs into the subject’s native space. Using this approach, each ROIs of the AAL template represented a node of the network.

To construct the interregional network, a DTI-based tractography method was used to generate brain white matter fiber tracts. For DTI preprocessing, diffusion MRI data were first register to non-diffusion-weighted images (b_0_ images) to correct eddy current distortion and head motion by using the EDDY and MCFLIRT function of Diffusion Toolbox of Functional MRI of the Brain (FMRIB) software [18]. A diffusion tensor model was then generated for each voxel to obtain three eigenvectors and three eigenvalues. Whole brain fiber tracking was further performed using the fiber assignment by continuous tracking (FACT) algorithm [19], starting from the center of each voxel and iterating along the main diffusion orientation of near voxels with a fractional anisotropy (FA) threshold of 0.2 and tracking turning angular threshold of 45°. In this process, a single seed was placed in the center of each voxel, and the path was continued into the adjacent voxel that minimized the path curvature. Paths were terminated for curvatures greater than 45° or FA less than 0.2. Fiber tracts that were rejected by the algorithm, such as those rejected due to high curvature, were not included in the present analysis.

### Tractography connectivity analysis

In tractography connectivity analysis, a graph [G = (V, E)] consists of a collection of nodes (V) and a collection of edges (E). To obtain regional structural connectivity matrix, a set of brain region masks representing V nodes, generated by previous anatomical image parcellation steps, was applied to the white matter tractography as represented by graph G, using the UCLA Multimodal Connectivity database [20]. For all pairs of brain regions, the number of white matter fiber tracts originating in one region (i) and terminating in another region (j) was counted and the fiber number was considered as the weight of each edge. After repeating this step for all regions in the template map, an interregional undirected weighted network with weighted connections between brain regions that are structurally connected was constructed.

The topology of the undirected weighted structural brain networks of LQS, HSD subtype and healthy controls was examined using graph theory. An individual’s weighted connectivity matrix M was first normalized to the maximum of M so that the overall differences in connectivity strength would be minimized. Then, for each weighted network connectivity matrix M, measures of small-world properties (i.e., clustering coefficient and characteristic shortest path length), and network efficiency (i.e., global efficiency and local efficiency), together with the feature of each node, including degree, clustering coefficient, betweenness centrality, and nodal efficiency, were computed with the Brain Connectivity Toolbox.

### ROI-based fractional anisotropy (FA) and mean diffusivity (MD) analysis

Diffusion tensor image reconstruction and postprocessing were performed using FMRIB’s Software Library. Fractional anisotropy (FA) and mean diffusivity (MD) were obtained using standard calculations in FMRIB’s Diffusion Toolbox [21]. After generating parametric maps of the DTI data, images of all the parametric maps were first linearly then non-linearly transformed to the MNI space. Then 90 cortical and subcortical ROIs were placed on the parametric maps to calculate the exact values.

### Statistical analysis

For baseline data, one-way analysis of variance (ANOVA) was used to analyze a difference in subjects’ age among the three groups, followed by Tukey’s method for further pairwise comparisons if the difference reached the significance level. Baseline continuous and categorical data between the two subtypes were analyzed using Student’s t-test Chi square (χ^2^) test, respectively.

For DTI data, all measures were averaged across the two hemispheres. There were a total of 276 DTI variables for statistical analysis as shown in Additional file 2: Table S1. Student’s t-test was applied for an initial screening. Those which showed potential statistical differences between the three groups were further analyzed using one-way analysis of covariance (ANCOVA) with covariates of age and gender, followed by Bonferroni correction t-test for pairwise comparisons if the difference reached significance. Linear regression was used to detect correlations between HAMD-24 score and DTI values in a pool of the LQS and HSD subtypes, and regression coefficients were obtained. Receiver operating characteristic (ROC) curve analysis was used to determine the cutoff values of DTI variables, at which LQS and HSD subtypes were dichotomized with acceptable sensitivity and specificity (defined as greater than 65% for both). Statistical significance was defined as a two-tailed P < 0.05. The analysis was conducted using SPSS v16.0 (SPSS Inc., Chicago, IL, USA).

## Results

### Participant characteristics

**Fig. 1:**
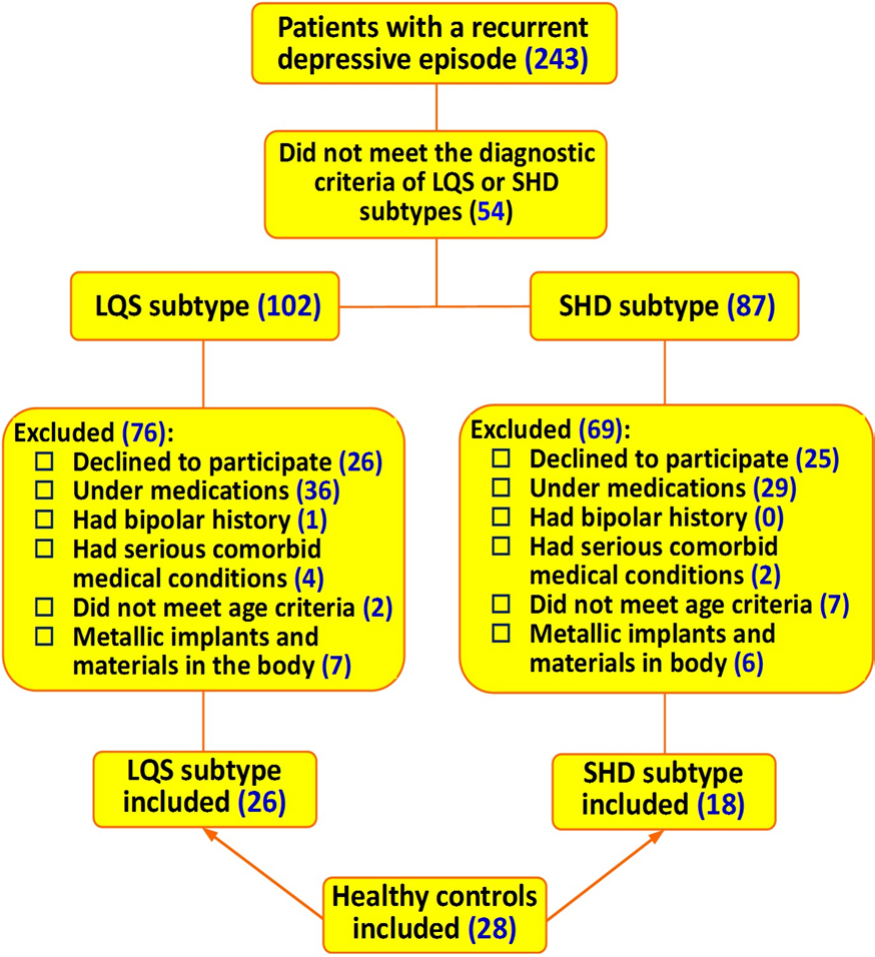
Recruitment profile. *LQS* liver qi stagnation, *HSD* heart spleen deficiency

Of the 243 patients who were currently experiencing a recurrent depressive episode, 102 and 87 met the diagnostic criteria of the LQS and SHD subtypes, respectively. Among them, 26 LQS subtypes and 18 SHD subtypes who met the inclusion criteria and agreed to participate in the study, and did not meet any exclusion criteria were recruited. Twenty-eight healthy volunteers were also recruited to serve as controls (Fig. 1). There were no significant differences in demographic and clinical variables among the three groups (Table 2).

**Table 2:** Demographic and clinical characteristics of participants

Variables	HC (n = 28)	LQS (n = 26)	HSD (n = 18)	*P* value
Male, n (%)^a^	11 (39.3)	5 (19.2)	9 (50.0)	0.088
Age (y)^b^	34.1 ± 8.1	39.5 ± 13.0	40.9 ± 10.0	0.107
Duration of the illness (months)^b^		34.2 ± 50.7	36.3 ± 49.7	0.890
Previous depressive episode^b^		1.8 ± 1.5	2.8 ± 3.7	0.214
HAMD-24 score^b^		32.3 ± 8.4	30.1 ± 6.9	0.365

*HC* healthy controls, *LQS* liver qi stagnation, *HSD* heart and spleen deficiency, *HAMD-24* 24-item Hamilton Rating Scale for Depression

^a^Categorical data was analyzed using Chi square test

^b^Continuous data are expressed mean ± SD and analyzed using Student t-test or one-way analysis of variance (ANOVA)

### Tractography connectivity variables

**Fig. 2:**
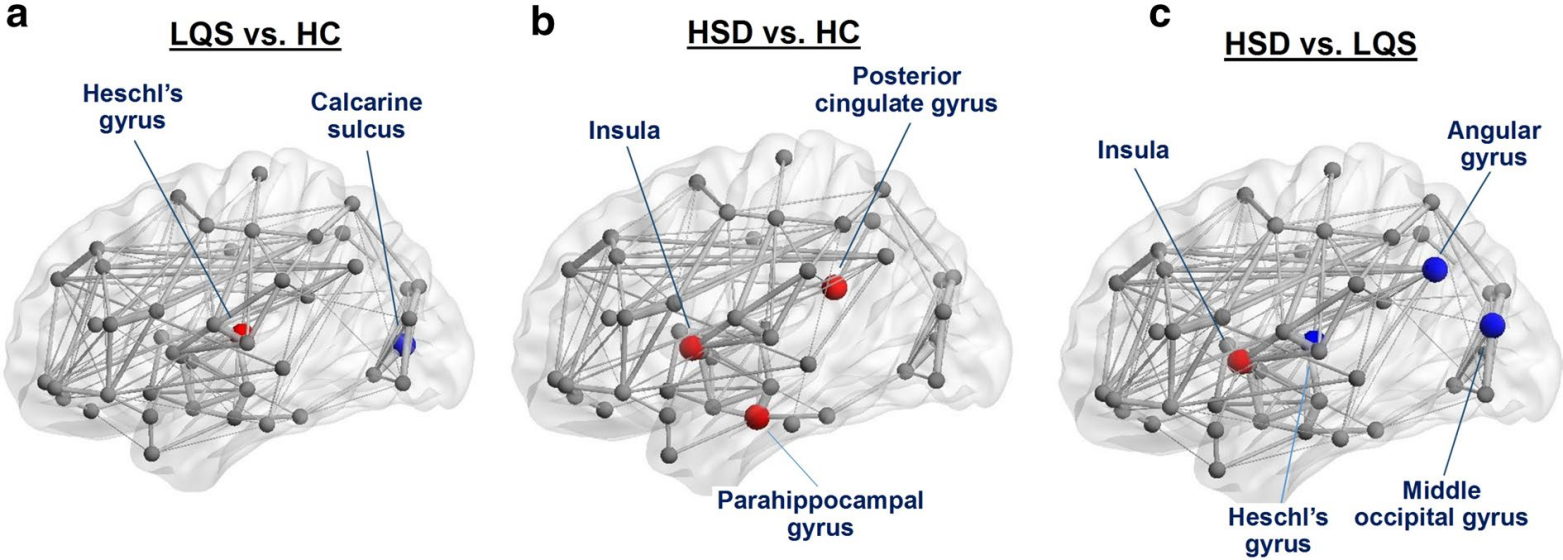
Diffusion tensor imaging based brain connectomic analysis. Liver qi stagnation (LQS) subtype versus healthy controls (HC) (**a**), Heart Spleen Deficiency (HSD) subtype versus HC (**b**), and HSD versus LQS (**c**). Red and blue filled circles indicate hyper- and hypo-connectivity, respectively, as compared with HC (**a**, **b**) or LQS (**c**)

ANCOVA analysis revealed significant differences in nodal degree in the calcarine sulcus and angular gyrus (*F*_2,68_ ≥ 3.584, *P* ≤ 0.033) (Table 3, Fig. 2). Pairwise comparisons further showed that the LQS subtype had a markedly lower degree than healthy controls in the calcarine sulcus (*P* < 0.001), but a significantly higher degree in the angular gyrus than the HSD subtype (*P* < 0.001).

**Table 3:** DTI-based connectomic analysis in patients with TCM-based subtypes of MDD

Brain regions	HC (n = 28)	LQS (n = 26)	HSD (n = 18)	ANCOVA		Bonferroni correction t-test		
				*F*_2,68_	*P*	HC/LQS	HC/HSD	HSD/LQS
Nodal degree								
Calcarine sulcus	27.3 ± 3.1	24.7 ± 2.9^#^	25.5 ± 3.2	3.584	0.033	< 0.001	0.279	0.451
Angular gyrus	11.7 ± 2.0	14.0 ± 3.9*	11.8 ± 2.7*	4.407	0.016	0.062	1.000	< 0.001
Betweenness centrality								
Insula	187.9 ± 142.5	231.5 ± 177.9*	349.7 ± 162.6^#^*	5.614	0.006	1.000	< 0.001	< 0.001
Posterior cingulate gyrus	138.6 ± 98.7	185.9 ± 148.0	232.8 ± 147.7^#^	3.555	0.034	0.480	< 0.001	0.131
Middle occipital gyrus	183.8 ± 161.5	287.9 ± 173.1*	185.6 ± 131.2*	3.556	0.034	0.094	1.000	0.003
Heschl’s gyrus	85.3 ± 61.1	166.1 ± 201.7^#^*	99.2 ± 102.5*	3.497	0.036	< 0.001	1.000	0.007
Clustering coefficient								
Parahippocampal gyrus	0.024 ± 0.009	0.027 ± 0.006	0.031 ± 0.011^#^	3.206	0.047	0.718	< 0.001	0.081
Fractional anisotropy (FA)								
Frontal medial orbital gyrus	0.217 ± 0.016	0.213 ± 0.020*	0.200 ± 0.015^#^*	4.580	0.014	1.000	< 0.001	< 0.001
Middle temporal pole	0.227 ± 0.015	0.228 ± 0.023*	0.215 ± 0.015^#^*	3.704	0.030	1.000	< 0.001	< 0.001
Mean diffusivity (MD)								
Frontal medial orbital gyrus	0.770 ± 0.034	0.767 ± 0.029*	0.794 ± 0.024^#^*	4.933	0.010	1.000	< 0.001	< 0.001

Data are expressed as mean ± SD. Analysis of covariance (ANCOVA) was used to detect significance, followed by Bonferroni correction t-test for pairwise comparisons: ^#^*P* < 0.05, vs. healthy controls; *P < 0.05, between the two subtypes. Those which *P* values examined with pairwise comparisons are highlighted as red fonts

*HC* healthy controls, *LQS* liver qi stagnation subtype, *HSD* heart and spleen deficiency subtype

Significant main effects on betweenness centrality (BC) were detected in the insula, posterior cingulate gyrus, middle occipital gyrus, and Heschl’s gyrus (*F*_2,68_ ≥ 3.497, *P* ≤ 0.036). The HSD subtype had greater BC values than healthy controls in the insula and posterior cingulate gyrus (*P* < 0.001). The BC value of the LQS subtype was significantly greater than that of the HSD subtype in the middle occipital gyrus (*P* = 0.003) and greater than those of the other two groups in Heschl’s gyrus (*P* ≤ 0.007), but markedly lower than that of the HSD subtype in the insula (P < 0.001).

Significant differences in clustering coefficient were observed in the parahippocampal gyrus (*F*_2,68_ = 3.206, *P* = 0.047). The coefficient of the HSD subtype was significantly greater than that of healthy controls in the parahippocampal gyrus (*P* < 0.001).

### FA and MD variables

**Fig. 3:**
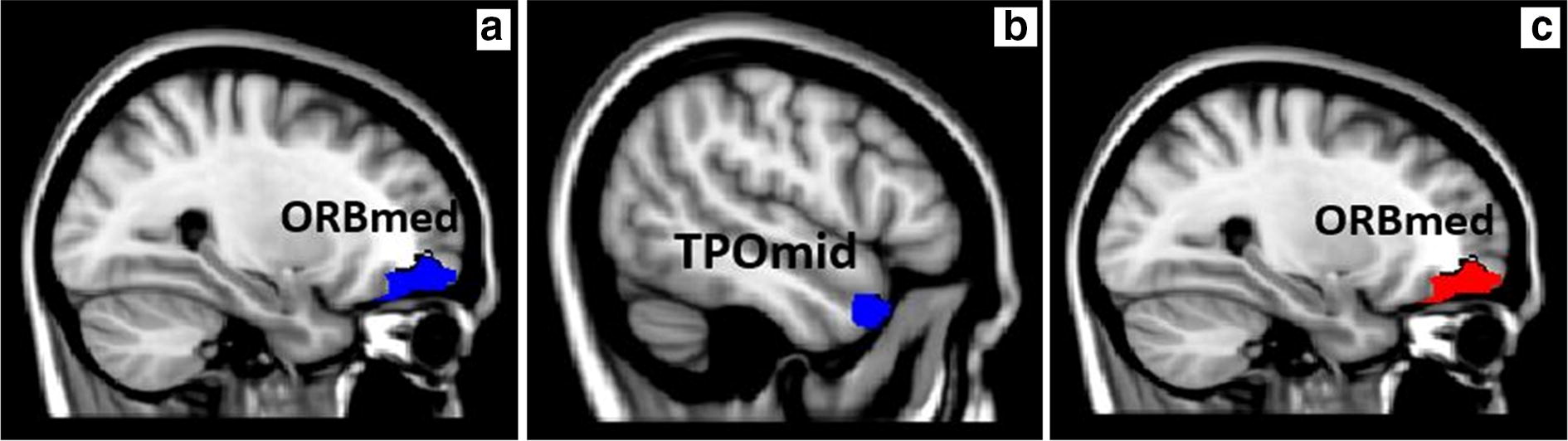
Diffusion tensor imaging white matter microstructural analysis. Significant decreases in fractional anisotropy (FA) of the frontal medial orbital gyrus (ORBmed, **a**) and the middle temporal pole (TPOmid, **b** indicated with blue) and significant increase in mean diffusivity (MD) of the frontal medial orbital gyrus (ORBmed, **c** indicated with red) in HSD subtype compared to the other two groups

ANCOVA analysis showed significant main effects on FA in the frontal medial orbital gyrus (*F*_2,68_ = 4.580, *P* = 0.014) and middle temporal pole (*F*_2,68_ = 3.704, *P* = 0.030), and MD in the frontal medial orbital gyrus (*F*_2,68_ = 4.933, *P* = 0.010) (Table 3, Fig. 3). Pairwise comparisons further revealed that the HSD subtype had significantly lower FA values in these two brain regions, but a markedly greater MD value in the medial orbital gyrus than the other two groups (*P* < 0.001).

### Correlations between HAMD score and DTI variables and ROC curve analysis

**Fig. 4:**
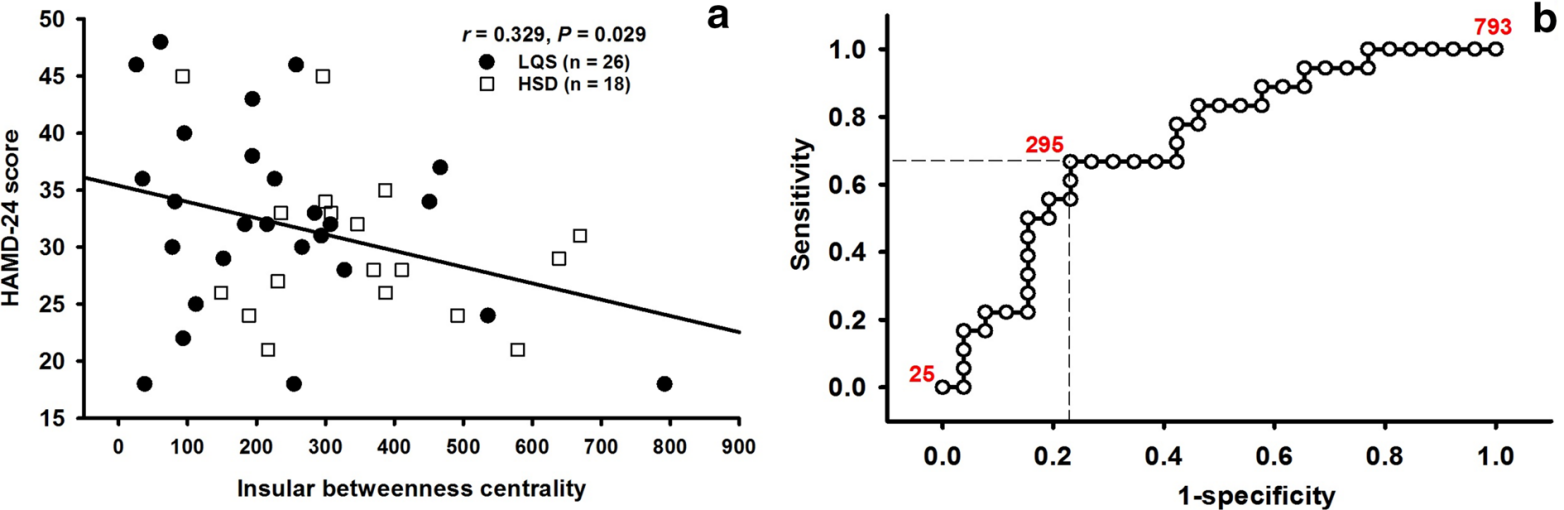
Insular betweenness centrality value is significantly inversely correlated with HAMD-24 score in a pool of liver qi stagnation (LQS) and heart spleen deficiency (HSD) subtypes (**a**). The two subtypes are well dichotomized at a cutoff value of 295 insular betweenness centrality with a sensitivity of 0.667 and a specificity of 0.769 (**b**)

When the LQS and HSD subtypes were pooled, significant correlation was only observed between the BC value of the insula and HAMD score in an inverse manner (r = 0.329, *P* = 0.029, n = 44) (Fig. 4a). ROC curve analysis further showed that both subtypes were well dichotomized at the cutoff value of 295 with a sensitivity of 0.667 and a specificity of 0.769 (Fig. 4b).

## Discussion

In parallel with our metabolomic findings [10], this study revealed differential brain connectomic profiles between the two subtypes of MDD. The principal findings of this study is that the LQS subtype is mainly characterized by aberrant connectivity of the audiovisual perception-related temporal-occipital network; whereas the HSD subtype is more closely associated with hyperconnectivity and microstructural abnormalities of the limbic-paralimbic network. In addition, we suggest that insular connectivity could serve a biomarker for TCM-based classification of depression.

We found that, compared to healthy controls and/or the HSD subtype, regional connectivity of the LQS subtype patients was increased in the angular gyrus, middle occipital gyrus, and Heschl’s gyrus, but decreased in the calcarine sulcus. The calcarine sulcus and occipital gyrus are directly involved in visual signal processing. Heschl’s gyrus, also known as the transverse temporal gyrus, processes incoming auditory signals. It therefore appears that the LQS subtype may represent aberrant connectivity of a temporal-occipital network involved in visual and auditory processing. Electrophysiological and fMRI studies have shown functional abnormalities in brain regions associated with visual attention, auditory processing, and cognitive control in depressed patients [22–24]. These findings could, at least in part, explain the fact that depressed individuals are characterized by biases in and ruminative responses to negative emotional materials [25], and the observation that visual and auditory hallucination often occurs in antidepressant treatment [26–30]. Clinically, the LQS subtype patients often exhibit stress-related comorbid symptoms and signs, such as nervousness, irritability, and pain disorders [6]. These may result from hypersensitivity or hyperarousal of an aberrant visual and auditory perception-related temporal-occipital network when these individuals are exposed to negative emotional circumstance.

In contrast, the HSD subtype displayed increased connectivity of multiple limbic and paralimbic regions, including the insula, posterior cingulate gyrus, and parahippocampal gyrus compared with healthy controls and/or the LQS subtype. One resting-state fMRI connectivity study has shown that patients with a TCM-based deficiency subtype of MDD exhibited increased connectivity of the posterior cingulate cortex with the bilateral middle frontal gyrus compared to healthy controls [9]. In this study, we further observed microstructural abnormality in the two paralimbic regions, the middle temporal pole, and the frontal medial orbital gyrus, as evidenced by the decreased FA values of both regions and the increased MD value of the latter region. It is suggested that the HSD subtype is mainly associated with hyperconnectivity and microstructural abnormalities of the limbic-paralimbic network. One recent meta-analysis has revealed hyperactivation in the subgenual anterior cingulate cortex and parahippocampal gyrus during affective processing tasks; but hypoactivation in the dorsal cingulate cortex and dorsal anterior insula during executive functioning tasks in youths with MDD compare to healthy controls [31]. The limbic and paralimbic system operates by influencing the endocrine system and the autonomic nervous system, and is heavily involved in emotional and cognitive processing [32, 33]. The hyperconnectivity of the limbic-paralimbic network observed in this study could be interpreted as evidence for the distinctive neurological, somatic, and cognitive symptoms which are often observed the HSD subtype. One fMRI study also has shown that a TCM-based deficiency subtype of MDD had markedly greater functional connectivity of the posterior cingulate cortex with the bilateral cerebellum and left superior frontal gyrus than the excess subtype [9].

This study further revealed a markedly negative correlation between insular BC value and the severity of depressive symptoms in a pool of the two subtypes of subjects. The optimal cutoff insular BC value dichotomized the two subtypes with acceptable sensitivity and specificity. These results suggest that insular connectivity may be a potential biomarker for differentiating the two subtypes of MDD. The insula is a critical neural substrate for integrating interoception into emotions, cognition, and decision-making [34, 35]. Abnormally increased functional neuroimaging activity of the anterior insula has been found in unmedicated subjects with primary MDD [36]. Insular hyper-reactivity has been implicated to be associated with negative overview on interoceptive states in depressed individuals [37]. In this study, insular hyperconnectivity was present in the HSD subtype, but not in the LQS subtype as compared with healthy subjects. This suggests a closer association of insular hyperconnectivity with the HSD subtype.

Our previous metabolomic study has suggested that, while both TCM subtypes are associated with aberrant proteinogenic branched-chain amino acid (BCAA) and energy metabolism, the LQS subtype is more closely associated with abnormalities in the biosynthesis of monoamine and amino acid neurotransmitters and has closer associations with stress-related pathophysiology [10]. On the other hand, MDD can be classified as ‘reactive’ or ‘endogenous’ (melancholic) subtypes on the basis of the presence or absence of traumatic stress prior to depressive onset [38]. Distinct changes in hippocampal genome-wide expression have been found in mouse models of ‘reactive’ and ‘endogenous’ subtypes [39]. The LQS subtype often manifests as an irritable, atypical depression with comorbid stress-related symptoms, including pain and digestive disorders; whereas the HSD subtype is often more closely associated with a ruminative subtype. Together with the current DTI results, it appears that the LQS subtype may represent a ‘reactive’ MDD subpopulation mainly characterized by monoamine and amino acid neurotransmitter abnormalities in stress processing-related temporal-occipital network; whereas the HSD subtype is related to ‘endogenous’ dysfunction of BCAA and energy metabolism, hyperconnectivity and microstructural abnormalities in the limbic-paralimbic network.

Several limitations of this study should be considered. First, the sample size of this study was relatively small. Similar flaws also have been widely observed in previous MRI connectivity studies of MDD [40]. In addition to insufficient statistical power to detect meaningful differences, small sample sizes may also result in heterogeneity across studies. Meta-analysis on a pool of connectomic studies of MDD and future large-scale research could yield more consistent and convincing results [40]. Second, although TCM diagnostic patterns have been well established in clinical practice, the procedure is largely based on empirical evidence rather than structured interview and well-designed instruments. This could cause “subjective” bias and deviation. In addition, while there are at least six subtypes of MDD according to TCM classification, this study only examined the two most opposing, commonly occurring subtypes [6–8]. Whether other subtypes have differential connectome profiles remains for further investigation. Third, we used DTI-based connectivity analysis, rather than histogram analysis. Although histogram analysis is an effective tool to detect and quantify early tissue changes in different diseases, it only has limited ability in linking anatomical changes with functional deficits [41]. In contrast, connectivity analysis is more powerful in drawing functional inferences from anatomical substrate [41]. Finally, this study was observational in nature. We did not examine whether the therapeutic response to antidepressants or non-pharmacological therapy, e.g., deep brain stimulation (DBT), is different between the two subtypes. Whether there exist TCM subtype-related differences in the antidepressant response deserves for further investigation.

## Conclusions

Collectively, the LQS subtype is mainly characterized by aberrant connectivity of the audiovisual perception-related temporal-occipital network, whereas the HSD subtype is more closely associated with hyperconnectivity and microstructural abnormalities of the limbic-paralimbic network. Insular connectivity is a potential biomarker for TCM diagnostic subtypes which is perhaps an alternative classification for depressive disorders.

## Additional files

**Additional file 1.** Minimum Standards of Reporting Checklist.

**Additional file 2: Table S1.** A statistical screening of connectomic variables between healthy subjects and traditional Chinese medicine diagnostic subtypes of major depressive disorders.

## Abbreviations

AAL: automated anatomical labeling
AC-PC: anterior commissure–posterior commissure
ANCOVA: analysis of covariance
ANOVA: analysis of variance
DSM-5: Statistical Manual of Mental Disorders, Fifth Edition
DTI: diffusion tensor imaging
FA: fractional anisotropy
FACT: fiber assignment by continuous tracking
FMRI: functional magnetic resonance imaging
FMRIB: functional MRI of the brain
FWHM: full width half maximum
HAMD-24: 24-item Hamilton Rating Scale for Depression
HSD: heart and spleen deficiency
LQS: liver qi stagnation
MD: mean diffusivity
MDD: major depressive disorder
MNI: Montreal Neurological Institute
MRI: magnetic resonance imaging
ROC: receiver operating characteristic
ROIs: regions of interest
SMP: statistical parametric mapping
TCM: traditional Chinese medicine
