# Supplementary material for: Brain connectomic associations with traditional Chinese medicine diagnostic classification of major depressive disorder: a diffusion tensor imaging study
Source: Chin Med. 2019 Apr 11;14:15. doi: 10.1186/s13020-019-0239-8 (PMC6460788; doi:10.1186/s13020-019-0239-8)
Supplement: Supplementary file 2 — Additional file 2: Table S1. A statistical screening of connectomic variables between healthy subjects and traditional Chinese medicine diagnostic subtypes of major depressive disorders. [file 13020_2019_239_MOESM2_ESM.pdf]

Table S1. A statistical screening of connectomic variables between healthy subjects and traditional Chinese medicine diagnostic subtypes of major depressive disorders

Variable	HC (n = 28)		LQS (n = 26)		HSD (n = 18)		*P* value of t-test [a]		
	Mean	SD	Mean	SD	Mean	SD	LQS/HC	HSD/HC	HSD/LQS
Overall measures									
Clustering coefficient	0.033	0.007	0.032	0.006	0.032	0.008	0.632	0.677	0.960
Shortest path length	75.972	3.069	74.946	2.584	75.314	2.908	0.191	0.473	0.661
Global efficiency	0.081	0.019	0.078	0.014	0.078	0.018	0.640	0.607	0.869
Local efficiency	0.063	0.014	0.059	0.010	0.060	0.014	0.348	0.494	0.961
Lambda	1.247	0.021	1.236	0.025	1.241	0.026	0.070	0.376	0.493
Gamma	2.230	0.125	2.268	0.164	2.322	0.165	0.346	0.038	0.288
Nodal Degree									
Precentral	20.643	2.802	20.250	3.004	19.444	2.332	0.621	0.139	0.345
Frontal Sup	26.446	3.074	25.519	4.433	24.944	3.531	0.373	0.134	0.649
Frontal Sup Orb	23.000	3.687	22.423	5.176	20.528	3.385	0.637	0.027	0.180
Frontal Mid	17.839	3.689	17.096	3.988	15.944	2.656	0.480	0.066	0.291
Frontal Mid Orb	15.018	2.837	14.385	4.788	12.833	2.854	0.554	0.015	0.226
Frontal Inf Oper	12.643	2.301	13.192	2.320	13.333	2.431	0.386	0.337	0.847
Frontal Inf Tri	18.929	3.661	19.635	4.151	19.111	3.483	0.510	0.867	0.663
Frontal Inf Orb	20.554	3.250	20.673	4.731	18.889	2.698	0.914	0.078	0.157
Rolandic Oper	13.179	2.195	14.423	2.576	13.417	1.629	0.061	0.695	0.151
Supp Motor Area	19.393	3.508	19.846	3.744	18.556	4.432	0.648	0.480	0.303
Olfactory	16.268	2.986	16.212	4.245	15.000	2.990	0.955	0.167	0.303
Frontal Sup Medial	22.143	3.786	21.692	4.596	20.861	2.950	0.695	0.230	0.503
Frontal Med Orb	18.339	3.852	17.769	4.723	18.333	3.831	0.628	0.996	0.677
Rectus	18.750	3.414	18.692	5.026	17.250	3.327	0.961	0.149	0.293
Insula	30.893	2.875	31.577	3.898	31.444	2.955	0.464	0.533	0.904
Cingulum Ant	18.589	2.957	18.250	3.272	18.083	2.891	0.691	0.571	0.863
Cingulum Mid	19.714	2.303	20.385	2.736	19.750	2.290	0.333	0.959	0.424
Cingulum Post_L	18.375	2.814	18.404	2.888	19.639	2.639	0.970	0.135	0.156
Hippocampus	34.357	3.106	34.019	4.691	34.139	3.451	0.755	0.825	0.927
Parahippocampal	22.107	2.511	21.365	4.614	20.639	5.067	0.462	0.197	0.624
Amygdala	18.804	2.760	17.212	3.835	17.861	2.817	0.084	0.268	0.544
Calcarine	27.286	3.056	24.667	2.910	25.538	3.234	0.006	0.046	0.365
Cuneus	22.786	2.970	20.442	3.772	21.472	4.157	0.014	0.218	0.398
Lingual	29.054	4.370	28.423	4.839	27.972	3.852	0.617	0.396	0.744
Occipital Sup	25.536	3.815	22.923	3.483	24.000	4.389	0.011	0.216	0.370
Occipital Mid	26.679	3.520	26.154	5.097	25.389	4.100	0.660	0.262	0.600
Occipital Inf	13.500	3.815	12.827	4.935	12.944	3.455	0.576	0.620	0.931
Fusiform	20.857	2.468	20.904	5.409	20.167	3.796	0.967	0.458	0.621
Postcentral	23.036	3.932	23.115	3.067	22.444	3.162	0.935	0.595	0.485
Parietal Sup	26.929	3.648	25.750	4.680	24.667	5.560	0.305	0.102	0.488
Parietal Inf	12.804	2.810	13.231	3.311	12.278	2.469	0.611	0.520	0.306
Supra Marginal	11.232	1.735	11.731	1.861	11.222	1.647	0.313	0.985	0.356
Angular	11.714	2.039	13.981	3.941	11.750	2.745	0.010	0.960	0.044
Precuneus	37.607	4.387	35.923	4.118	36.889	2.983	0.153	0.546	0.399
Paracentral Lobule	14.661	2.611	15.750	3.418	14.194	2.590	0.192	0.556	0.110
Caudate	28.589	3.664	30.077	3.506	28.361	3.189	0.134	0.830	0.105
Putamen	33.732	3.668	33.577	3.851	33.083	3.602	0.880	0.559	0.670

Pallidum	20.018	3.213	20.538	2.839	21.167	3.808	0.532	0.277	0.534
Thalamus	27.554	5.120	26.635	4.538	25.778	4.466	0.490	0.235	0.539
Heschl	9.304	2.339	9.981	2.326	9.278	2.123	0.291	0.970	0.313
Temporal Sup	22.071	2.751	23.212	3.587	22.750	2.840	0.194	0.424	0.651
Temporal Pole Sup	22.250	4.022	20.712	4.260	20.278	2.824	0.178	0.077	0.708
Temporal Mid	25.464	3.426	24.058	2.418	24.194	3.582	0.089	0.235	0.880
Temporal Pole Mid	16.500	3.618	15.385	3.067	15.806	3.344	0.229	0.517	0.668
Temporal Inf	21.429	3.182	20.423	5.155	18.917	5.068	0.389	0.044	0.343
Between centrality									
Precentral	144.33	118.79	130.47	106.70	93.89	95.27	0.655	0.137	0.250
Frontal Sup	238.40	161.04	159.50	91.36	203.84	134.64	0.033	0.454	0.199
Frontal Sup Orb	175.98	141.09	225.19	150.36	171.74	149.45	0.220	0.923	0.252
Frontal Mid	201.85	186.39	139.39	139.20	135.58	114.00	0.171	0.183	0.924
Frontal Mid Orb	136.59	98.97	171.54	131.59	141.74	137.32	0.273	0.883	0.472
Frontal Inf Oper	37.29	50.96	55.56	58.51	70.74	113.92	0.226	0.180	0.565
Frontal Inf Tri	140.35	139.72	125.04	94.84	110.93	71.58	0.642	0.414	0.596
Frontal Inf Orb	253.89	200.38	214.23	132.12	182.20	93.43	0.398	0.163	0.381
Rolandic Oper	75.12	81.45	78.36	83.12	67.25	69.16	0.885	0.737	0.644
Supp Motor Area	169.46	147.83	205.86	149.80	185.10	104.31	0.373	0.698	0.614
Olfactory	277.44	216.78	343.92	182.52	285.07	231.31	0.230	0.910	0.351
Frontal Sup Medial	206.99	168.65	209.22	114.44	180.84	129.31	0.955	0.579	0.447
Frontal Med Orb	150.59	94.12	162.94	129.30	168.12	145.05	0.688	0.621	0.902
Rectus	242.66	197.92	206.93	164.64	165.91	123.38	0.476	0.149	0.375
Insula	187.94	142.54	231.48	177.91	349.72	162.56	0.324	0.001	0.030
Cingulum Ant	183.08	112.56	179.84	159.44	152.39	95.04	0.931	0.344	0.517
Cingulum Mid	211.45	146.20	143.43	101.23	149.05	90.22	0.054	0.112	0.851
Cingulum Post_L	138.65	98.70	185.86	147.96	232.84	147.70	0.171	0.013	0.306
Hippocampus	387.12	236.63	330.84	226.78	459.06	243.40	0.377	0.325	0.081
Parahippocampal	338.94	209.28	253.69	166.09	226.10	159.68	0.105	0.058	0.585
Amygdala	219.16	114.65	198.34	141.91	284.27	210.91	0.554	0.182	0.113
Calcarine	217.27	173.24	179.33	147.65	188.36	157.49	0.392	0.570	0.847
Cuneus	210.18	134.63	227.56	181.15	167.08	110.25	0.689	0.263	0.214
Lingual	254.89	145.98	320.56	215.91	331.60	204.82	0.193	0.145	0.866
Occipital Sup	175.85	133.33	204.74	150.73	230.91	198.54	0.458	0.266	0.622
Occipital Mid	183.77	161.48	287.92	173.15	185.61	131.18	0.026	0.968	0.040
Occipital Inf	107.83	99.54	95.73	105.21	77.81	84.52	0.666	0.296	0.552
Fusiform	157.47	139.37	158.17	133.11	151.48	137.19	0.985	0.887	0.872
Postcentral	190.11	134.10	179.18	146.63	169.74	102.57	0.776	0.586	0.815
Parietal Sup	237.22	123.88	260.41	154.03	301.18	195.95	0.543	0.181	0.444
Parietal Inf	84.09	89.98	97.70	99.45	117.97	128.56	0.600	0.298	0.559
Supra Marginal	21.57	33.23	37.60	43.29	26.28	36.70	0.131	0.655	0.370
Angular	79.10	75.98	72.38	80.86	67.72	60.00	0.754	0.594	0.836
Precuneus	297.87	141.86	349.77	183.69	387.56	196.66	0.249	0.079	0.518
Paracentral Lobule	131.25	133.71	148.23	88.52	108.63	94.63	0.588	0.536	0.163
Caudate	375.17	200.39	359.49	144.30	432.67	175.53	0.744	0.325	0.138
Putamen	341.79	224.66	367.72	216.35	323.96	169.48	0.668	0.775	0.477
Pallidum	213.74	163.83	272.53	208.93	278.15	218.77	0.253	0.260	0.932
Thalamus	429.51	271.90	349.59	232.24	400.06	182.97	0.253	0.688	0.445
Heschl	85.33	61.11	99.21	102.47	166.14	201.69	0.545	0.032	0.109

Temporal Sup	153.24	107.02	192.50	156.74	190.97	117.29	0.284	0.267	0.972
Temporal Pole Sup	268.63	179.13	205.02	126.63	180.68	110.97	0.141	0.069	0.514
Temporal Mid	146.15	117.31	149.64	107.84	163.93	112.02	0.910	0.612	0.673
Temporal Pole Mid	108.07	98.00	117.60	88.06	125.94	129.34	0.710	0.597	0.800
Temporal Inf	160.58	113.16	150.04	82.46	180.46	192.80	0.699	0.661	0.477
Nodal clustering coefficient									
Precentral	0.039	0.014	0.045	0.012	0.040	0.011	0.092	0.641	0.225
Frontal Sup	0.026	0.008	0.030	0.009	0.028	0.012	0.136	0.626	0.502
Frontal Sup Orb	0.022	0.009	0.022	0.008	0.023	0.007	0.809	0.600	0.432
Frontal Mid	0.036	0.015	0.041	0.013	0.038	0.015	0.138	0.637	0.382
Frontal Mid Orb	0.020	0.008	0.023	0.006	0.022	0.008	0.295	0.449	0.890
Frontal Inf Oper	0.055	0.027	0.061	0.021	0.054	0.019	0.358	0.880	0.257
Frontal Inf Tri	0.037	0.012	0.040	0.015	0.038	0.010	0.296	0.642	0.569
Frontal Inf Orb	0.027	0.012	0.027	0.006	0.029	0.009	0.896	0.528	0.437
Rolandic Oper	0.042	0.023	0.044	0.014	0.041	0.014	0.738	0.867	0.518
Supp Motor Area	0.027	0.009	0.029	0.008	0.028	0.008	0.365	0.815	0.531
Olfactory	0.014	0.007	0.012	0.004	0.015	0.007	0.172	0.711	0.080
Frontal Sup Medial	0.026	0.010	0.026	0.007	0.027	0.007	0.865	0.806	0.622
Frontal Med Orb	0.023	0.008	0.024	0.007	0.026	0.008	0.657	0.412	0.607
Rectus	0.019	0.008	0.019	0.006	0.021	0.007	1.000	0.392	0.341
Insula	0.031	0.012	0.032	0.007	0.029	0.007	0.588	0.480	0.101
Cingulum Ant	0.022	0.008	0.024	0.007	0.022	0.008	0.307	0.910	0.421
Cingulum Mid	0.026	0.009	0.028	0.008	0.026	0.010	0.254	0.983	0.331
Cingulum Post_L	0.029	0.011	0.026	0.008	0.025	0.008	0.335	0.194	0.559
Hippocampus	0.026	0.009	0.026	0.006	0.027	0.007	0.923	0.677	0.672
Parahippocampal	0.024	0.009	0.027	0.006	0.031	0.012	0.170	0.038	0.197
Amygdala	0.019	0.007	0.021	0.007	0.022	0.008	0.275	0.166	0.627
Calcarine	0.035	0.013	0.035	0.009	0.038	0.011	0.840	0.409	0.438
Cuneus	0.035	0.011	0.033	0.010	0.036	0.014	0.687	0.750	0.524
Lingual	0.028	0.008	0.030	0.006	0.030	0.007	0.476	0.349	0.670
Occipital Sup	0.033	0.012	0.033	0.009	0.034	0.011	0.880	0.845	0.697
Occipital Mid	0.030	0.013	0.031	0.007	0.032	0.010	0.950	0.609	0.516
Occipital Inf	0.036	0.014	0.042	0.020	0.043	0.017	0.215	0.114	0.794
Fusiform	0.040	0.015	0.044	0.018	0.047	0.018	0.363	0.134	0.549
Postcentral	0.033	0.012	0.037	0.011	0.035	0.012	0.285	0.663	0.594
Parietal Sup	0.026	0.010	0.027	0.006	0.026	0.007	0.752	0.971	0.693
Parietal Inf	0.051	0.021	0.057	0.020	0.052	0.024	0.255	0.825	0.452
Supra Marginal	0.060	0.021	0.061	0.018	0.062	0.019	0.779	0.731	0.915
Angular	0.055	0.021	0.051	0.018	0.053	0.018	0.470	0.721	0.756
Precuneus	0.026	0.009	0.027	0.005	0.027	0.008	0.401	0.663	0.767
Paracentral Lobule	0.025	0.010	0.030	0.011	0.028	0.009	0.069	0.315	0.453
Caudate	0.020	0.007	0.020	0.005	0.020	0.006	0.955	0.929	0.958
Putamen	0.023	0.008	0.025	0.006	0.024	0.007	0.282	0.712	0.516
Pallidum	0.020	0.007	0.022	0.006	0.021	0.007	0.243	0.678	0.569
Thalamus	0.021	0.007	0.024	0.005	0.022	0.008	0.100	0.570	0.436
Heschl	0.026	0.009	0.028	0.008	0.026	0.011	0.404	0.973	0.510
Temporal Sup	0.034	0.013	0.035	0.009	0.034	0.011	0.753	0.955	0.700
Temporal Pole Sup	0.025	0.011	0.029	0.007	0.033	0.014	0.150	0.037	0.185
Temporal Mid	0.036	0.013	0.039	0.009	0.039	0.014	0.284	0.444	0.958

Temporal Pole Mid	0.030	0.012	0.034	0.010	0.039	0.018	0.209	0.042	0.211
Temporal Inf	0.036	0.012	0.041	0.013	0.047	0.021	0.149	0.036	0.275
Temporal Inf	0.063	0.020	0.070	0.017	0.077	0.028	0.193	0.056	0.291
Fractional anisotropy (FA)									
Precentral	0.231	0.014	0.231	0.012	0.234	0.019	0.977	0.553	0.548
Frontal Sup	0.216	0.012	0.215	0.014	0.216	0.018	0.808	0.975	0.838
Frontal Sup Orb	0.262	0.019	0.261	0.022	0.256	0.020	0.757	0.322	0.522
Frontal Mid	0.206	0.012	0.203	0.013	0.205	0.015	0.352	0.848	0.563
Frontal Mid Orb	0.225	0.015	0.220	0.017	0.222	0.017	0.275	0.592	0.678
Frontal Inf Oper	0.235	0.014	0.233	0.017	0.233	0.016	0.632	0.666	0.989
Frontal Inf Tri	0.236	0.016	0.233	0.015	0.233	0.019	0.429	0.634	0.872
Frontal Inf Orb	0.219	0.018	0.217	0.012	0.212	0.012	0.749	0.192	0.174
Rolandic Oper	0.214	0.014	0.214	0.015	0.208	0.020	0.920	0.261	0.321
Supp Motor Area	0.205	0.013	0.207	0.014	0.208	0.014	0.751	0.598	0.820
Olfactory	0.255	0.038	0.252	0.032	0.253	0.034	0.748	0.872	0.895
Frontal Sup Medial	0.191	0.010	0.190	0.011	0.188	0.015	0.595	0.441	0.739
Frontal Med Orb	0.217	0.016	0.213	0.020	0.200	0.015	0.400	0.001	0.031
Rectus	0.255	0.026	0.252	0.021	0.253	0.026	0.628	0.767	0.907
Insula	0.222	0.012	0.221	0.013	0.219	0.015	0.735	0.447	0.647
Cingulum Ant	0.190	0.014	0.191	0.013	0.192	0.017	0.834	0.768	0.894
Cingulum Mid	0.204	0.010	0.207	0.013	0.207	0.019	0.212	0.501	0.846
Cingulum Post_L	0.419	0.027	0.416	0.031	0.416	0.038	0.745	0.751	0.963
Hippocampus	0.298	0.019	0.293	0.021	0.291	0.022	0.424	0.235	0.657
Parahippocampal	0.233	0.016	0.234	0.017	0.227	0.014	0.797	0.202	0.157
Amygdala	0.256	0.020	0.256	0.023	0.258	0.028	0.996	0.825	0.840
Calcarine	0.226	0.012	0.226	0.015	0.220	0.017	0.846	0.221	0.216
Cuneus	0.199	0.013	0.203	0.012	0.199	0.011	0.202	0.911	0.181
Lingual	0.215	0.014	0.215	0.010	0.211	0.012	0.789	0.402	0.199
Occipital Sup	0.231	0.014	0.241	0.018	0.235	0.016	0.023	0.357	0.256
Occipital Mid	0.228	0.014	0.232	0.010	0.226	0.012	0.316	0.585	0.106
Occipital Inf	0.223	0.017	0.224	0.012	0.219	0.012	0.907	0.374	0.221
Fusiform	0.226	0.014	0.228	0.016	0.222	0.015	0.636	0.354	0.207
Postcentral	0.211	0.012	0.215	0.012	0.213	0.015	0.255	0.627	0.668
Parietal Sup	0.191	0.011	0.196	0.014	0.191	0.015	0.151	0.968	0.254
Parietal Inf	0.193	0.011	0.195	0.015	0.189	0.017	0.612	0.338	0.244
Supra Marginal	0.205	0.010	0.203	0.011	0.201	0.017	0.453	0.285	0.599
Angular	0.227	0.013	0.228	0.010	0.223	0.013	0.696	0.253	0.100
Precuneus	0.220	0.010	0.217	0.010	0.218	0.013	0.311	0.546	0.826
Paracentral Lobule	0.206	0.014	0.204	0.018	0.202	0.017	0.682	0.370	0.670
Caudate	0.317	0.026	0.316	0.025	0.318	0.028	0.907	0.912	0.833
Putamen	0.345	0.020	0.351	0.025	0.353	0.027	0.402	0.314	0.812
Pallidum	0.348	0.039	0.339	0.036	0.332	0.034	0.393	0.159	0.496
Thalamus	0.394	0.016	0.403	0.030	0.395	0.026	0.179	0.865	0.384
Heschl	0.168	0.014	0.172	0.020	0.168	0.017	0.352	0.890	0.520
Temporal Sup	0.202	0.012	0.202	0.015	0.195	0.012	0.904	0.061	0.100
Temporal Pole Sup	0.188	0.018	0.186	0.017	0.178	0.012	0.588	0.030	0.090
Temporal Mid	0.234	0.012	0.232	0.013	0.226	0.012	0.622	0.034	0.115
Temporal Pole Mid	0.227	0.015	0.228	0.023	0.215	0.015	0.850	0.013	0.041
Temporal Inf	0.258	0.012	0.255	0.014	0.249	0.016	0.486	0.059	0.228

Mean diffusivity (MD)									
Precentral	0.743	0.027	0.742	0.034	0.747	0.029	0.882	0.640	0.602
Frontal Sup	0.785	0.024	0.779	0.026	0.778	0.026	0.408	0.321	0.818
Frontal Sup Orb	0.749	0.030	0.739	0.039	0.754	0.030	0.271	0.632	0.185
Frontal Mid	0.768	0.028	0.766	0.026	0.759	0.032	0.710	0.302	0.459
Frontal Mid Orb	0.793	0.025	0.794	0.031	0.791	0.029	0.949	0.765	0.752
Frontal Inf Oper	0.762	0.019	0.756	0.021	0.749	0.028	0.350	0.074	0.323
Frontal Inf Tri	0.748	0.020	0.745	0.021	0.743	0.030	0.664	0.540	0.781
Frontal Inf Orb	0.770	0.020	0.767	0.020	0.766	0.024	0.553	0.524	0.886
Rolandic Oper	0.762	0.017	0.762	0.020	0.762	0.026	0.946	0.956	0.999
Supp Motor Area	0.764	0.020	0.759	0.019	0.756	0.026	0.393	0.261	0.646
Olfactory	0.770	0.021	0.774	0.020	0.768	0.028	0.395	0.761	0.342
Frontal Sup Medial	0.788	0.020	0.780	0.024	0.779	0.022	0.232	0.174	0.838
Frontal Med Orb	0.770	0.033	0.767	0.029	0.794	0.024	0.696	0.014	0.002
Rectus	0.769	0.027	0.763	0.021	0.768	0.034	0.391	0.946	0.541
Insula	0.790	0.016	0.787	0.015	0.787	0.022	0.502	0.651	0.956
Cingulum Ant	0.812	0.016	0.809	0.016	0.809	0.021	0.523	0.518	0.891
Cingulum Mid	0.787	0.018	0.783	0.015	0.783	0.022	0.387	0.588	0.900
Cingulum Post_L	0.750	0.017	0.751	0.018	0.751	0.020	0.898	0.905	0.993
Hippocampus	0.816	0.018	0.812	0.021	0.816	0.023	0.478	0.920	0.518
Parahippocampal	0.792	0.023	0.790	0.020	0.797	0.022	0.692	0.453	0.245
Amygdala	0.795	0.027	0.788	0.038	0.794	0.035	0.492	0.938	0.630
Calcarine	0.810	0.017	0.808	0.014	0.813	0.014	0.567	0.556	0.220
Cuneus	0.808	0.017	0.804	0.016	0.809	0.016	0.377	0.849	0.310
Lingual	0.794	0.018	0.791	0.014	0.796	0.014	0.423	0.680	0.206
Occipital Sup	0.800	0.018	0.791	0.022	0.799	0.020	0.096	0.820	0.224
Occipital Mid	0.798	0.020	0.792	0.017	0.794	0.017	0.283	0.562	0.684
Occipital Inf	0.792	0.022	0.786	0.017	0.791	0.019	0.336	0.935	0.391
Fusiform	0.786	0.019	0.783	0.021	0.791	0.026	0.566	0.495	0.285
Postcentral	0.757	0.024	0.750	0.028	0.761	0.027	0.348	0.577	0.203
Parietal Sup	0.757	0.036	0.756	0.027	0.747	0.043	0.893	0.370	0.376
Parietal Inf	0.805	0.021	0.802	0.024	0.800	0.023	0.593	0.441	0.803
Supra Marginal	0.802	0.021	0.803	0.019	0.802	0.021	0.913	0.963	0.884
Angular	0.791	0.025	0.790	0.021	0.780	0.021	0.852	0.121	0.132
Precuneus	0.780	0.022	0.779	0.017	0.782	0.017	0.865	0.739	0.566
Paracentral Lobule	0.723	0.030	0.719	0.030	0.731	0.030	0.566	0.401	0.184
Caudate	0.727	0.025	0.728	0.021	0.733	0.026	0.774	0.381	0.486
Putamen	0.700	0.021	0.702	0.022	0.705	0.031	0.719	0.472	0.670
Pallidum	0.678	0.039	0.690	0.032	0.697	0.057	0.223	0.177	0.588
Thalamus	0.692	0.020	0.688	0.024	0.692	0.027	0.541	0.950	0.591
Heschl	0.806	0.025	0.796	0.024	0.795	0.029	0.181	0.192	0.848
Temporal Sup	0.778	0.017	0.775	0.018	0.778	0.022	0.557	0.985	0.630
Temporal Pole Sup	0.775	0.027	0.774	0.029	0.778	0.034	0.888	0.768	0.694
Temporal Mid	0.774	0.017	0.773	0.018	0.774	0.020	0.847	0.974	0.848
Temporal Pole Mid	0.783	0.025	0.781	0.037	0.793	0.031	0.830	0.200	0.239
Temporal Inf	0.761	0.021	0.761	0.023	0.763	0.027	0.957	0.763	0.747

a. The highlighted variables which showed potential statistical differences among the three groups were further analyzed using analysis of covariance (ANCOVA) and are summarized in Table 3. HC, Healthy controls; LQS, Liver Qi stagnation subtype; HSD, Heart and Spleen Deficiency subtype.
